# Bibliometric analysis of neural injury biomarkers in neurodegenerative diseases: research trends and future perspectives

**DOI:** 10.3389/fnhum.2025.1614132

**Published:** 2025-06-18

**Authors:** Chengzhou Pa, Shijun Shen, Yunrui Dai, Min Wu

**Affiliations:** ^1^Department of Hepatobiliary Pancreatic and Vascular Surgery, First People’s Hospital of Kunming, The Affiliated Calmette Hospital of Kunming Medical University, Kunming, China; ^2^Department of Minimally Invasive Hepatobiliary and Pancreatic Surgery, Lincang People's Hospital, Lincang, China; ^3^Department of Magnetic Resonance Imaging, First People’s Hospital of Yunnan Province, Affiliated Hospital of Kunming University of Science and Technology, Kunming, China; ^4^Department of Oncology, Third People’s Hospital of Honghe Prefecture, Gejiu, China

**Keywords:** neural injury biomarkers, neurodegenerative diseases, multimodal biomarkers, citation analysis, bibliometric analysis

## Abstract

**Introduction:**

Neurodegenerative diseases (NDs) are progressive disorders with an increasing global health impact. Neural injury biomarkers have emerged as potential tools for early diagnosis and disease monitoring.

**Methods:**

To map research trends in this field, we conducted a comprehensive bibliometric analysis of 1,228 peer-reviewed articles published from 1991 to 2024 using CiteSpace and the Bibliometrix R package.

**Results:**

Our analysis revealed steady publication growth, particularly accelerating after 2015. The United States, United Kingdom, and China produced the highest volume of publications and citations, with institutions such as the University of California System and Harvard University serving as key collaboration hubs. Early research prioritized tau, amyloid-beta (Aβ), cerebrospinal fluid (CSF), and mild cognitive impairment (MCI). Since 2020, the focus has expanded to blood-based biomarkers, exosomal microRNAs, and inflammation-related markers such as glial fibrillary acidic protein (GFAP) and translocator protein (TSPO). Through citation and clustering analyses, we identified three developmental phases: (1) CSF-based amyloid/tau validation, (2) multimodal and genetic biomarker integration, and (3) the emergence of plasma and neuroinflammatory markers.

**Discussion:**

These trends reflect a paradigm shift toward minimally invasive and multifactorial diagnostic approaches. Our findings underscore evolving priorities in NDs biomarker research and highlight the importance of multi-omics, artificial intelligence (AI), and interdisciplinary collaboration for translational discovery and clinical application.

## 1 Introduction

Neurodegenerative diseases (NDs), including Alzheimer’s disease (AD), Parkinson’s disease (PD), and frontotemporal dementia (FTD), are defined by progressive neuronal degeneration, frequently accompanied by cognitive decline and motor dysfunction (Dugger and Dickson, 2017). With aging populations worldwide, the number of individuals with dementia is projected to reach 152.8 million by 2050 (Collaborators, 2022), of which AD accounts for approximately 70% of cases (Reitz and Mayeux, 2014). This escalation poses substantial societal and economic burdens globally. While advancements in genetics and neuroimaging have enhanced our understanding of NDs pathophysiology, the lack of definitive, non-invasive diagnostic tools remains a critical barrier to early detection and disease-modifying therapies.

A key challenge in managing NDs is the absence of reliable biomarkers for early diagnosis (Logroscino et al., 2022). Emerging evidence highlights neural injury biomarkers—encompassing proteins (Raghunathan et al., 2022), lipids (Wei et al., 2023), biofluids (Alcolea et al., 2023), and imaging markers (Dilliott et al., 2023)—as pivotal indicators of neuroinflammation, oxidative stress (OS), and neurodegeneration. Despite their promise, gaps persist in translating these biomarkers into clinical practice (Cheslow et al., 2024). Major hurdles include standardizing protocols for sample collection, improving assay reproducibility, and establishing population-specific cutoff thresholds. Additionally, limited disease specificity restricts the clinical utility of many biomarkers. Addressing these challenges is essential to bridge the gap between discovery and clinical implementation.

Bibliometric analysis has proven effective for mapping research trends, identifying key contributors, and forecasting emerging topics (Hicks et al., 2015; Wei et al., 2022). Although prior studies have broadly examined NDs, few focus specifically on neural injury biomarkers. This study aims to provide a comprehensive bibliometric analysis of research trends, key contributors, and future directions in the application of neural injury biomarkers for NDs.

## 2 Data and methods

### 2.1 Retrieval strategy and data collection

We extracted data from the Web of Science (WOS) Core Collection database, selected for its coverage of over 12,000 academic journals and frequent use in prior bibliometric studies (Ege et al., 2023; Jiang et al., 2023; Wang et al., 2024). Our search strategy targeted studies on neural injury biomarkers in NDs, filtering for “Article” and “Review” document types in English (1991–2024; search date: December 11, 2024). Data were exported in plain text format. The search strategy is detailed in Table 1. A topic search (TS) was conducted, encompassing titles, abstracts, and keywords. The search query combined terms via Boolean operators: TS = (“Neural injury biomarkers” OR “Neurological biomarkers” OR NSE OR S100B OR GFAP OR Tau OR UCH-L1) AND (Neurodegenerative diseases OR “Alzheimer’s disease” OR “Parkinson’s disease” OR “Huntington’s disease” OR “Amyotrophic lateral sclerosis” OR “Multiple sclerosis”). A total of 1,228 articles were retrieved for analysis.

**Table 1:** Literature search strategy used in the web of science core collection.

Search phase	Search strategies	Literature number
#1	TS = (“Neural injury biomarkers” OR “Neurological biomarkers” OR NSE OR S100B OR GFAP OR Tau OR UCH-L1)	176001
#2	TS = (Neurodegenerative diseases OR “Alzheimer’s disease” OR “Parkinson’s disease” OR “Huntington’s disease” OR “Amyotrophic lateral sclerosis” OR “Multiple sclerosis”)	44232
#3	#1 and #2	1228

Data retrieved from web of science core collection (1991–2024). Search conducted on December 11, 2024.

### 2.2 Inclusion and exclusion criteria

Inclusion criteria: studies categorized as “Article” or “Review” in the WOS database and addressing the application of neural injury biomarkers in NDs.

Exclusion criteria: articles excluded from the analysis included: (1) conference papers, newspapers, books, and other non-peer-reviewed sources; (2) duplicate records; (3) articles with incomplete bibliographic information.

### 2.3 Data analysis

Bibliometric analysis was performed using CiteSpace v.6.2. R4 (64-bit) and the “Bibliometrix” R package. CiteSpace is a software tool for visualizing and analyzing scientific literature (Chen, 2004). It enables exploration of citation networks, keyword co-occurrence, author collaboration patterns, and the identification of highly-cited references and keywords experiencing citation bursts over specific periods. These features assist in understanding research trends, hotspots, and the evolution of scientific fields. Bibliometrix is an open-source, free-to-use tool written in R (Aria and Cuccurullo, 2017). The Bibliometrix R package was used to extract core bibliometric elements, including titles, abstracts, authors, references, institutions, countries, and keywords. It also generates graphical representations that facilitate the comprehensive analysis of the literature’s knowledge structure.

To ensure reproducibility and comparability, all analyses were conducted using default settings. In CiteSpace, the g-index algorithm was applied with a scaling factor of k = 25. CiteSpace used spectral clustering combined with modularity optimization (resolution parameter *γ* = 1.0, default) to identify network communities. Clusters were labeled using the log-likelihood ratio (LLR) algorithm, which prioritizes statistically significant and distinctive terms.

## 3 Results

### 3.1 Overview of main information

**Figure 1:**
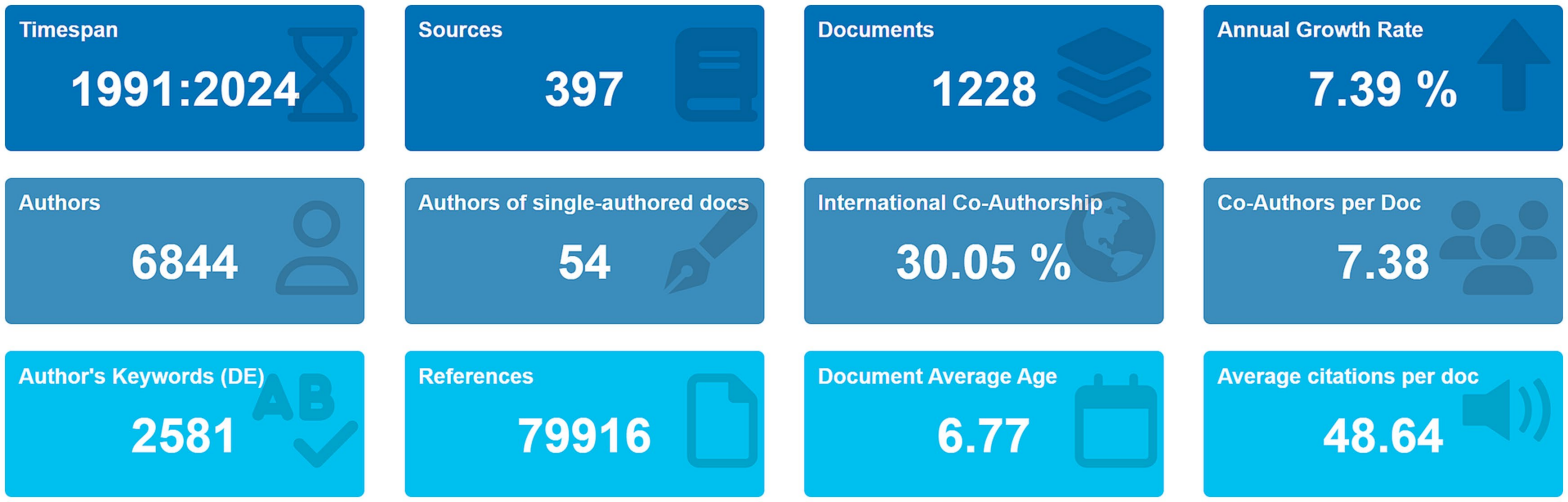
Bibliometric landscape of neurodegenerative disease biomarker research from 1991 to 2024.

Analysis of the WOS Core Collection data (Figure 1) revealed 1,228 articles published between 1991 and 2024 across 397 journals. Among these, 834 (67.92%) were original research articles, while 394 (32.08%) were reviews. The field exhibited an average annual growth rate of 7.39%, with contributions from 6,844 authors and international collaboration in 30.05% of publications. The total citation count reached 79,916, yielding an average of 48.64 citations/article.

### 3.2 Publication output trends

**Figure 2:**
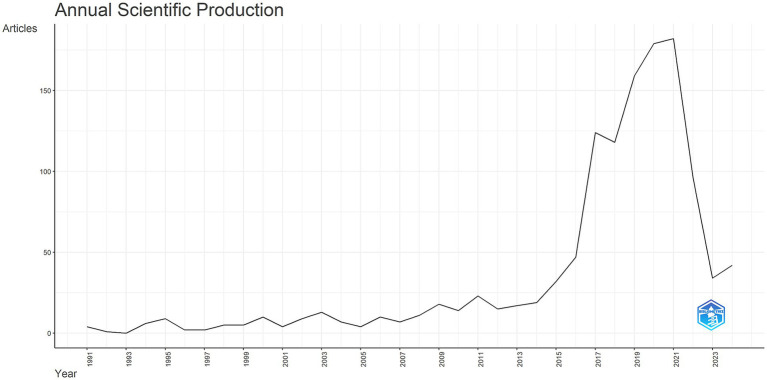
Annual scientific production in neurodegenerative disease biomarker research (1991–2024).

The trends in publication output reveal notable shifts in research focus over time (Figure 2). Between 1991 and 2015, publications on neural injury biomarkers remained stable. However, a marked increase in the volume of publications was observed from 2015 onward, with a peak of 188 articles published in 2020. This surge correlated with the advent of high-sensitivity assays such as single-molecule array (SIMOA) and increased funding for translational research following failed anti-amyloid drug trials. These trends underscore growing global interest in biomarkers for NDs diagnosis and prognosis.

### 3.3 Analysis of countries, institutions, and authors

**Figure 3:**
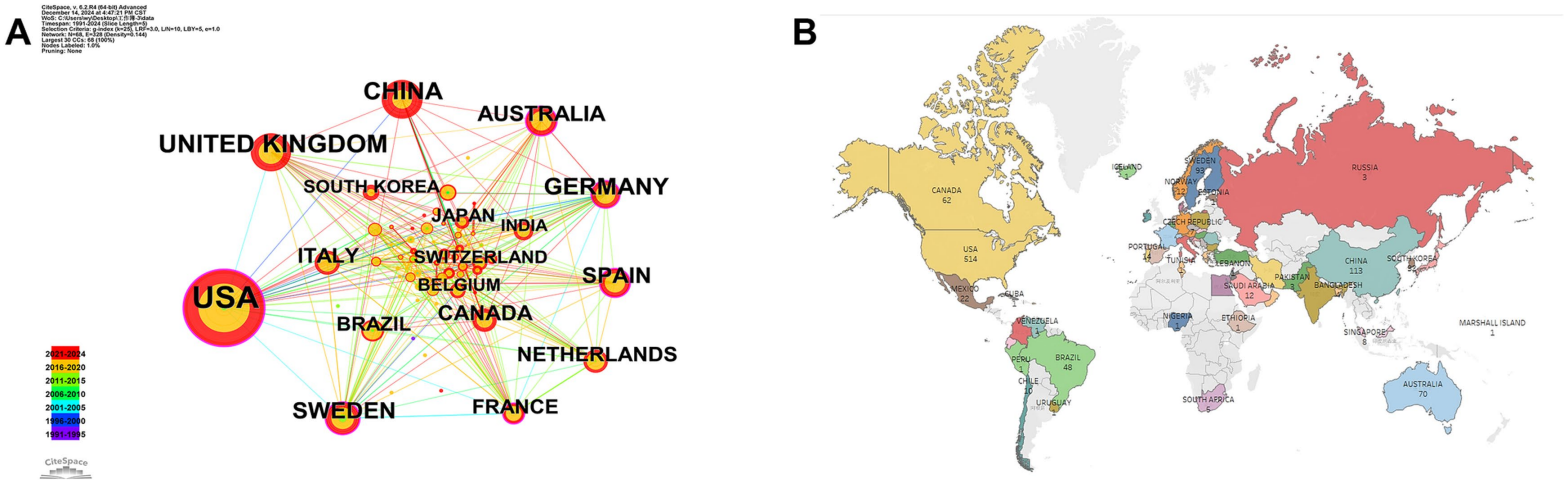
Geographic distribution and international collaboration in neurodegenerative disease biomarker research. **(A)** Country collaboration network. **(B)** Global publication distribution by country.

As shown in Figures 3A,B, the United States contributed the largest number of publications (514 articles, 41.86%), followed by the United Kingdom (136 articles, 11.07%), China (113 articles, 9.20%), Germany (108 articles, 8.79%), and Sweden (93 articles, 7.57%). These findings are further supported by Table 2, which shows the United States, Sweden, and China as the leading countries in terms of citation counts. This suggests that these countries are major drivers of research in the field of neural injury biomarkers for NDs.

**Table 2:** Citation metrics by country in neurodegenerative disease biomarker research.

Rank	Country	Total citations (TC)	Average article citations
1	USA	21,882	53.50
2	Sweden	4,970	146.20
3	China	3,698	33.00
4	United Kingdom	3,507	41.30
5	Brazil	3,294	78.40
6	Germany	3,019	48.70
7	Australia	2,041	44.40
8	Canada	1,992	45.30
9	France	1,979	79.20
10	Italy	1,691	51.20

**Figure 4:**
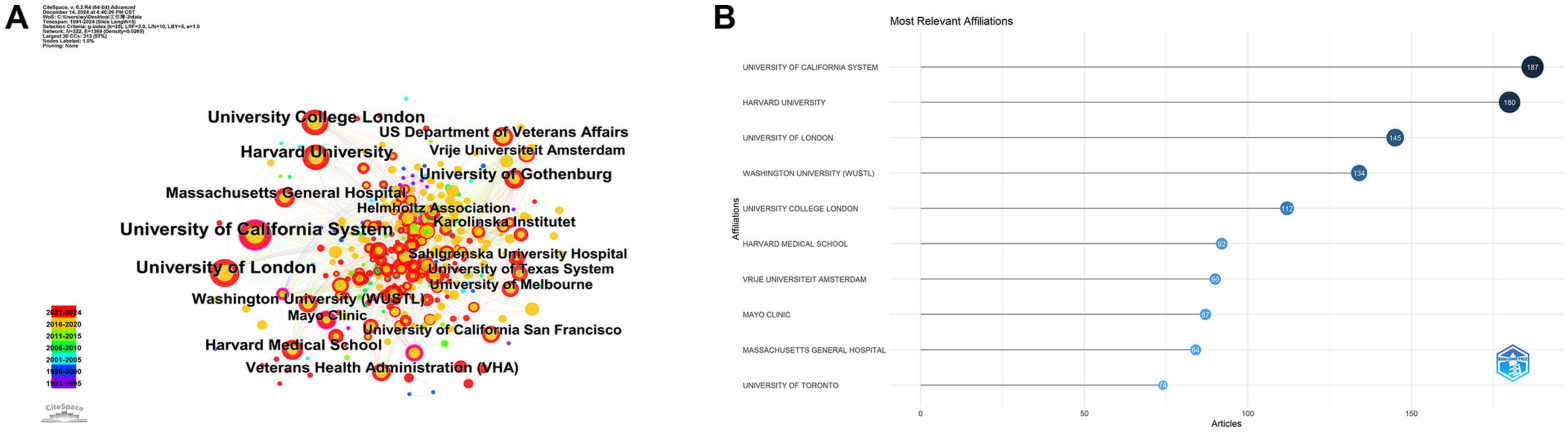
Institutional contributions. **(A)** Co-authorship network among institutions. **(B)** Top contributing institutions by number of publications.

The leading institutions in this area of research include the University of California System (187 articles), Harvard University (180 articles), University of London (145 articles), Washington University (134 articles), and University College London (112 articles) (Figures 4A,B). These institutions have made significant contributions to the field, reflecting their role as key research hubs for neural injury biomarkers.

**Figure 5:**
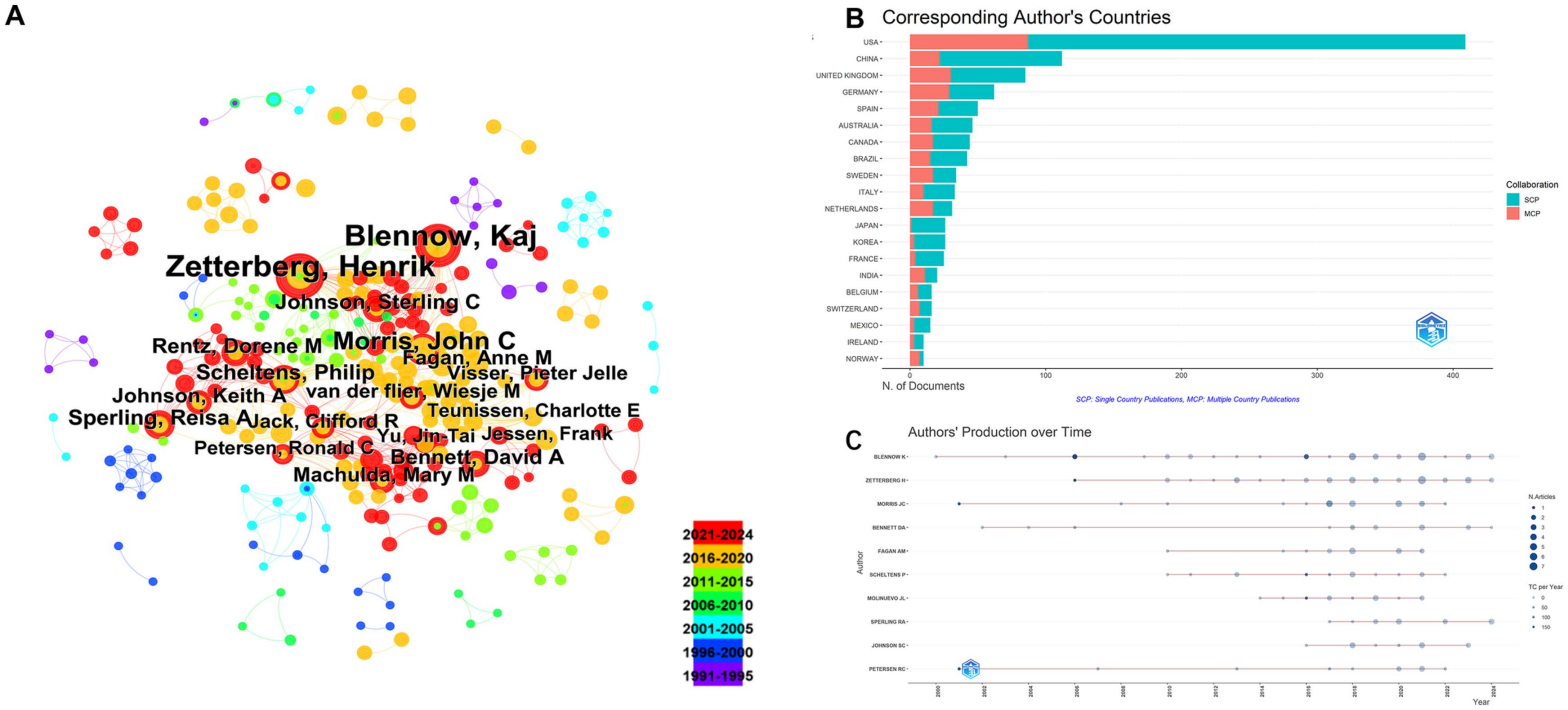
Author-level collaboration and productivity analysis. **(A)** Co-authorship network among authors. **(B)** Distribution of corresponding authors’ countries. **(C)** Temporal distribution of author productivity.

These institutions have made significant contributions to the field, underscoring their role as central research hubs for neural injury biomarkers. The analysis of corresponding authors’ countries (Table 3) reveals that the United States leads with the highest percentage of single-country publications (78.7%), while Germany stands out for its higher proportion of multi-country publications (46.8%). This highlights the strong international collaborative nature of research in this domain. Figure 5A demonstrates that Blennow K and Zetterberg H are two of the most active authors in this field, consistently contributing to the literature over time. Their substantial output reflects their critical role in advancing the understanding of NDs. Figure 5B further emphasizes the geographic distribution of research activity, with the United States, China, and the United Kingdom leading in terms of research output. Notably, Germany and Sweden exhibit a high degree of international collaboration, with over 40% of their publications resulting from multi-country partnerships, reflecting the global interconnectedness of research efforts. As shown in Figure 5C, the production of these prominent authors has remained consistent over the years, with annual publications continuing to reflect their sustained influence and contributions to the field.

**Table 3:** Country composition of corresponding authors in neurodegenerative disease biomarker research.

Rank	Country	Articles	Percentage (%)	SCP	MCP	MCP %
1	USA	409	33.3	322	87	21.3
2	China	112	9.1	90	22	19.6
3	United Kingdom	85	6.9	55	30	35.3
4	Germany	62	5	33	29	46.8
5	Spain	50	4.1	29	21	42
6	Australia	46	3.7	30	16	34.8
7	Canada	44	3.6	27	17	38.6
8	Brazil	42	3.4	27	15	35.7
9	Sweden	34	2.8	17	17	50
10	Italy	33	2.7	23	10	30.3

SCP: Publications involving authors from a single country; MCP: Publications involving authors from multiple countries.

### 3.4 Core journals

**Figure 6:**
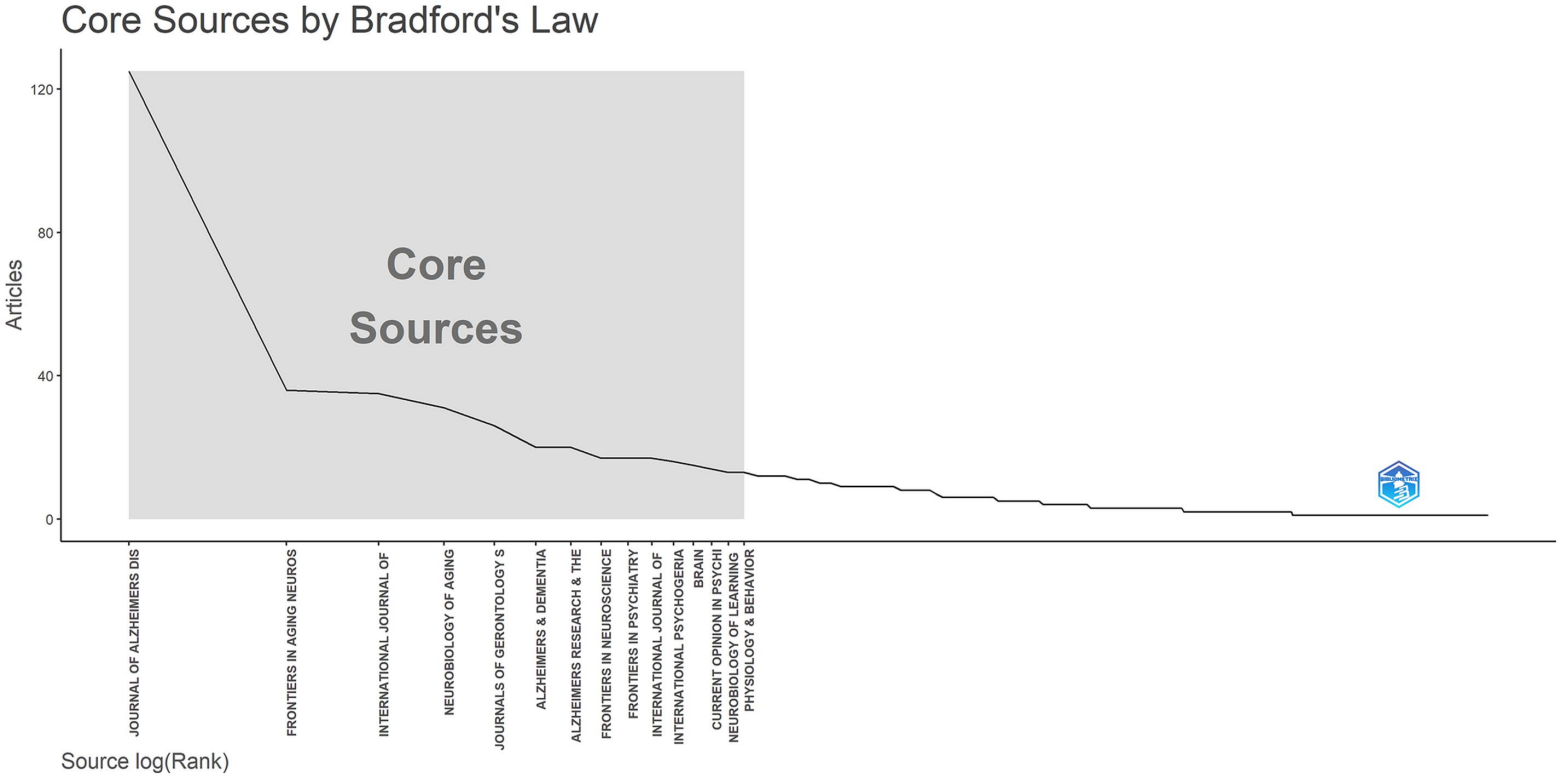
Core journals in neural injury biomarker research: a Bradford’s law analysis.

Based on Bradford’s Law, 15 core journals were identified in this field (Figure 6 and Table 4). The top five journals contributing the most to this research area were the Journal of AD, Frontiers in Aging Neuroscience, International Journal of Geriatric Psychiatry, Neurobiology of Aging, and Journals of Gerontology Series A: Biological Sciences and Medical Sciences. These journals have become crucial platforms for publishing groundbreaking studies in the field.

**Table 4:** Core journals identified in neurodegenerative disease biomarker research according to Bradford’s Law.

Rank	Journal	Frequency	Cumulative Frequency
1	Journal of Alzheimers Disease	125	125
2	Frontiers in Aging Neuroscience	36	161
3	International Journal of Geriatric Psychiatry	35	196
4	Neurobiology of Aging	31	227
5	Journals of Gerontology Series A-Biological Sciences And Medical Sciences	26	253
6	Alzheimers and Dementia	20	273
7	Alzheimers Research and Therapy	20	293
8	Frontiers in Neuroscience	17	310
9	Frontiers in Psychiatry	17	327
10	International Journal of Molecular Sciences	17	344
11	International Psychogeriatrics	16	360
12	Brain	15	375
13	Current Opinion in Psychiatry	14	389
14	Neurobiology of Learning and Memory	13	402
15	Physiology and Behavior	13	415

### 3.5 Keyword frequency and research hotspots

**Figure 7:**
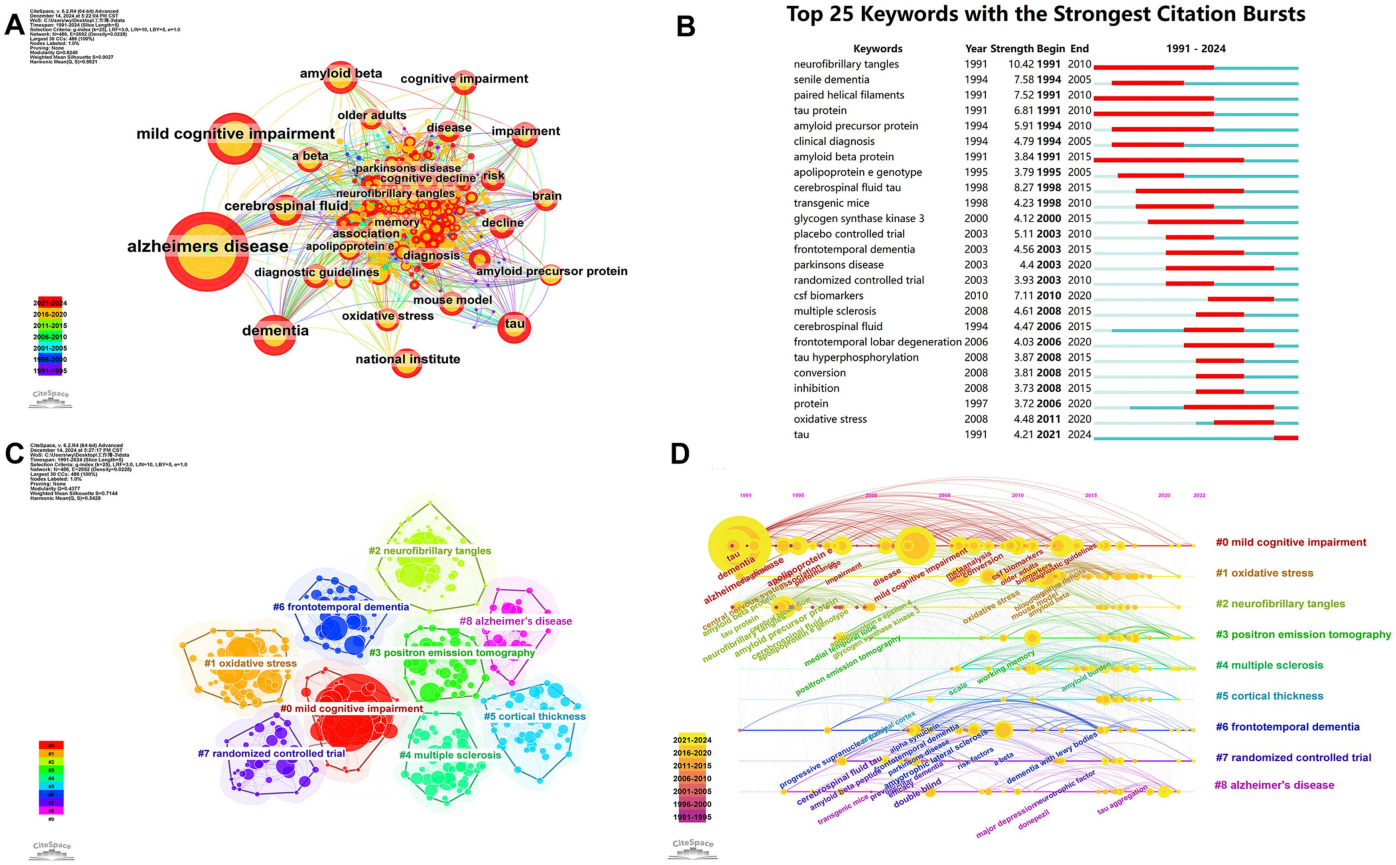
Evolution of research hotspots and thematic trends in neurodegenerative disease biomarker research. **(A)** Keyword co-occurrence network. **(B)** Top 25 keywords with the strongest citation bursts. **(C)** Keyword clusters based on log-likelihood ratio (LLR) analysis. **(D)** Timeline visualization of keyword cluster development (1991–2024).

The frequency of keywords was analyzed to identify research hotspots. The co-occurrence network (Figure 7A) highlighted prominent topics such as AD, mild cognitive impairment (MCI), dementia, amyloid beta (Aβ), cerebrospinal fluid (CSF), tau, and the National Institute. Figure 7B displays the top 25 keywords with the strongest citation bursts, with neurofibrillary tangles (NFTs) exhibiting the highest burst strength (10.42). Prior to 2015, research focused on senile dementia, FTD, and multiple sclerosis (MS), with an emphasis on randomized controlled trials, transgenic mouse studies, tau protein, amyloid precursor protein (APP), and apolipoprotein E (*APOE*) genotype. However, after 2015, there was a shift toward PD, frontotemporal lobar degeneration (FTLD), and CSF biomarkers. From 2021 to 2024, the keyword “tau” remained a focal point. Figure 7C illustrates the nine most representative clusters, with a Modularity Q value of 0.44, indicating strong clustering, and a Weighted Mean Silhouette S value of 0.71, signifying a compelling cluster structure. These clusters included MCI, OS, NFTs, positron emission tomography (PET), MS, cortical thickness, FTD, randomized controlled trials, and AD. Figure 7D illustrates the development and transformation of keywords in each cluster. It allows us to better recognize changes in a particular topic in a research field over time and to quickly understand the development and frontiers of the field.

**Figure 8:**
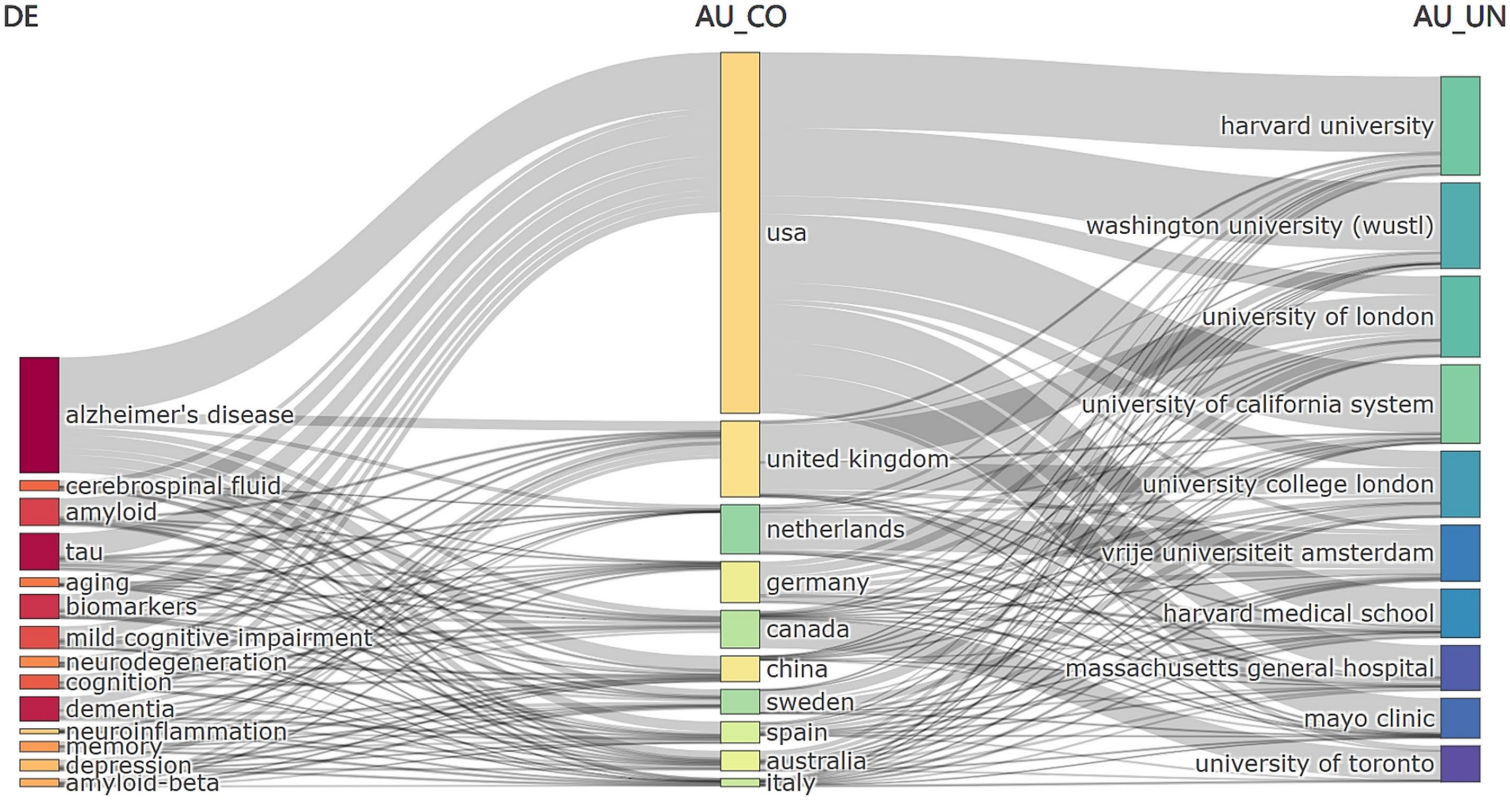
Interlinkages among keywords, author countries, and institutions in neurodegenerative disease biomarker research.

Additionally, Figure 8 shows the link between keywords (left), authors’ nationalities (center) and institutions (right). The area of the rectangle is proportional to the number of publications and shows that AD major researchers are from the United States and that multiple institutions are involved in the field.

### 3.6 Co-cited authors and literature

Table 5 lists the top 10 most co-cited articles. Among the most co-cited authors, Petersen et al. (2001) (Mayo Clinic Rochester, USA) had the highest co-citation count (3,719), establishing it as the most influential study in this field. Other highly co-cited works include those by Blennow et al. (2006) (Sahlgren’s University Hospital, Sweden; citation count: 3,358) and Dubois et al. (2016) (Sorbonne Universities, France; citation count: 1,261). These studies have made seminal contributions to understanding neural injury biomarkers in NDs.

**Table 5:** Top 10 most co-cited articles in the field of neurodegenerative disease biomarkers.

Rank	References (author, year)	Journal (abbreviation)	Article highlights	TC	TC per year	Normalized TC
1	Petersen et al. (2001)	Arch Neurol-Chicago	Defined MCI as a transitional stage between aging and AD, emphasizing early biomarkers	3719	148.76	3.93
2	Blennow et al. (2006)	Lancet	Comprehensive review of Aβ and tau pathology in AD	3358	167.90	6.51
3	Dubois et al. (2016)	Alzheimers Dement	Advocated preclinical CSF biomarkers for clinical trials	1261	126.10	16.89
4	Bennett et al. (2006)	Lancet Neurol	Linked social engagement to delayed cognitive decline	511	25.55	0.99
5	Guo et al. (2020)	Mol Neurodegener	Proposed novel diagnostic strategies for Aβ and tau pathologies	506	84.33	12.55
6	Silva et al. (2019)	J Biomed Sci	Analyzed modifiable risk factors for AD prevention	470	67.14	9.37
7	Schliebs and Arendt (2006)	J Neural Transm	Explored cholinergic dysfunction in AD	439	21.95	0.85
8	Liu et al. (2019)	Signal Transduct Tar	Synthesized hypotheses for AD therapeutic trials	434	62.00	8.65
9	Arendt et al. (2016)	Brain Res Bull	Discussed molecular mechanisms of tau pathology	406	40.60	5.44
10	Kirova et al. (2015)	Biomed Res Int	Advocated neuropsychological testing for MCI detection	390	35.45	7.85

TC, Total Citations; Normalized TC, Citations adjusted for publication year.

## 4 Discussion

This study presents the comprehensive bibliometric analysis focused specifically on neural injury biomarkers in NDs. It identifies evolving research priorities from CSF-based biomarkers to minimally invasive blood-derived markers, especially post-2020. Notably, the study highlights emerging interest in neuroinflammation and OS as integral pathological components. Furthermore, it delineates three distinct developmental stages of the field, offering a temporal framework that may guide future research and clinical translation.

### 4.1 Overview of development in the field

The publication volume has increased steadily over the past three decades, with a pronounced surge post-2015. This growth aligns with rising global interest in biomarkers for neurodegeneration and their potential to improve diagnostic and prognostic accuracy. The United States, United Kingdom, and China emerged as leading contributors in both publication output and citation impact.

The most frequently studied biomarkers included tau protein, Aβ, CSF biomarkers, and NFTs. These results corroborate established knowledge linking tau (Scheltens et al., 2021; Ossenkoppele et al., 2022) and Aβ (Soderberg et al., 2023; Jia et al., 2024) to AD pathophysiology. However, our analysis revealed a paradigm shift toward OS-and neuroinflammation-related biomarkers post-2015. Although OS and neuroinflammation represent distinct pathological processes, they exhibit bidirectional interactions: OS triggers reactive oxygen species (ROS) production, leading to lipid peroxidation, protein misfolding, and DNA damage, while chronic neuroinflammation exacerbates neuronal injury via microglial activation (Islam, 2017; Singh et al., 2019; Teleanu et al., 2022). This shift underscores the multifactorial nature of NDs and the need for biomarkers capturing broader pathological spectra.

### 4.2 Analysis of countries, institutions, and journals

From the perspective of international cooperation and research teams, scientific research institutions in the United States, the United Kingdom, China, Germany and other countries dominate the field, forming several research teams with international influence. The relatively close cooperation network among these teams has contributed to the rapid development of research in this field. However, we also note that despite the significant increase in research results in this field, there are still many challenges in the clinical translational application of biomarkers, such as the specificity, sensitivity, and standardization of biomarkers, which still need to be further researched and solved. Future research should emphasize multidisciplinary integration and foster tighter connections between basic and clinical research to enhance the real-world utility of neural injury biomarkers. Research in this area comes from the University of California System, Harvard University, University of London, Washington University, and University College London.

Moreover, our analysis identified key journals such as Journal of AD and Frontiers in Aging Neuroscience as central to the dissemination of research on neural injury biomarkers. These journals have become crucial platforms for publishing groundbreaking studies and significantly contributing to the field’s development.

### 4.3 Research hotspots and trends

Keyword co-occurrence analysis identified nine core clusters with AD MCI and Aβ/tau pathology forming recent investigative axes. MCI affects 20–50% of older adults and represents a prodromal stage where early intervention may delay dementia progression (Chaudhary et al., 2020; Tabeeva, 2019). The Montreal Cognitive Assessment (MoCA) demonstrates 89% sensitivity and 75% specificity outperforming traditional tools (Tan and Tan, 2021).

Plasma homocysteine (Hcy) and APOE ε4 are associated with the risk of NDs, especially in patients with AD and MCI, with patients with non-carrier genotypes showing more pronounced changes in the neurodegenerative marker (phosphorylated tau 217) (Lin et al., 2025). In addition, retinal imaging, a non-invasive modality, has also shown promise in detecting early microvascular and structural alterations linked to MCI (Christinaki et al., 2022).

OS and neuroinflammation, as intertwined mechanisms, drive lipid peroxidation, protein aggregation, and DNA damage, culminating in neuronal apoptosis (Maciejczyk et al., 2020). Elevated OS markers such as 8-hydroxydeoxyguanosine (8-OHdG) correlate with cognitive decline in AD (Graille et al., 2020; Ghezzi and Mooradian, 2021), while ROS overproduction activates pro-inflammatory cytokines (IL-1β, TNF-*α*), amplifying neuroinflammation via microglial activation (Dhapola et al., 2021; Leng and Edison, 2021). These findings support antioxidant and anti-inflammatory therapies for disease mitigation.

NFTs, beyond influencing neuronal survival, correlate with pathological progression. Hippocampal NFTs density inversely correlates with cognitive impairment in AD (Iliyasu et al., 2023), while tauopathy-specific NFTs distribution aids differential diagnosis (Richardson et al., 2024). Tau-specific PET radiotracers (e.g., ^18^F-THK5351, ^18^F-MK-6240) enable *in vivo* NFTs quantification with 85–90% accuracy (Ishibashi et al., 2024), and transcriptomics link NFTs formation to neuronal stress responses (Otero-Garcia et al., 2022). While PET achieves 90% amyloid detection accuracy, its accessibility remains limited (Doruyter et al., 2021). Conversely, blood-based biomarkers [e.g., plasma neurofilament light chain (NfL) (Quiroz et al., 2020), p-tau217 (Ashton et al., 2022)] revolutionize scalable screening. Emerging frontiers include FTD-specific TAR DNA-binding protein 43 (TDP-43) biomarkers (Chatterjee et al., 2024) and randomized controlled trial (RCT) designs for anti-amyloid therapy evaluation (Hoilund-Carlsen et al., 2024; Logroscino et al., 2025).

This study delineates three transformative phases in neural injury biomarker research, each demarcated by pivotal external events that reshaped the trajectory of biomarker development: (1) 1991–2015: Amyloid/tau hypothesis validation; (2) 2015–2020: Multimodal biomarker integration; (3) 2020–2024: Emergence of blood-based biomarkers and neuroinflammation focus.

#### 4.3.1 Phase I (1991–2015): amyloid and tau hypothesis validation

Foundational work validated the amyloid cascade hypothesis, focusing on CSF Aβ42 and tau detection. Hardy and Higgins established Aβ’s role in AD progression (Hardy and Higgins, 1992), while Blennow et al. (2006) demonstrated diagnostic utility of CSF p-tau/Aβ42 ratios. Despite APP/PS1 transgenic models linking amyloid pathology to cognitive deficits (Jankowsky et al., 2003), clinical translation faced challenges due to CSF invasiveness and poor early-stage correlation (Jack, 2012). Growing skepticism toward amyloid-centric therapies emerged as clinical benefits remained elusive.

#### 4.3.2 Phase II (2015–2020): multimodal biomarker integration, prompted by trial failures

The failure of bapineuzumab Phase 3 trials (Salloway et al., 2014) prompted a paradigm shift toward multimodal integration. MTBR-tau243 in CSF correlated strongly with tau-PET (r = 0.83), surpassing conventional p-tau markers (Horie et al., 2023). Genetic risk factors (APOE ε4, TREM2) gained traction as prognostic indicators (Yeh et al., 2017; Jia et al., 2020). The SIMOA platform achieved 0.62 pg./mL sensitivity, exceeding ELISA and electrochemiluminescence (ECL) assays (Kuhle et al., 2016).

#### 4.3.3 Phase III (2020–2024): rise of blood biomarkers and focus on neuroinflammation

Recent advancements emphasize blood-based biomarkers and neuroinflammation. Plasma p-tau181 reduces tau-PET screening failures by ~50% (Moscoso et al., 2022), while plasma glial fibrillary acidic protein (GFAP) mediates Aβ-PET effects on tau-PET burden and cognitive decline (Pereira et al., 2021). Blood exosomes reflect amyloid and NFTs pathology (Liu et al., 2022), and translocator protein (TSPO) PET maps neuroinflammation *in vivo* (De Picker et al., 2023). While PET radio-ligands provide regional specificity, integrating fluid-based biomarkers—such as CSF cytokines or plasma GFAP—with neuroimaging endophenotypes offers unprecedented insights into the spatiotemporal interplay between neuroinflammation and AD pathophysiology (Hampel et al., 2020). The ATI(N) framework incorporates inflammation into the amyloid/tau/neurodegeneration (ATN) scheme (Lista et al., 2024), enabling multidimensional assessment via PET, blood biomarkers, and machine learning.

### 4.4 Future directions

Integrating neuroinflammation and OS into biomarker panels is critical. Plasma GFAP and soluble TREM2 reflect astrocytic and microglial activity, respectively, while 8-OHdG and F2-isoprostanes are emerging peripheral neuronal injury markers. Composite panels (e.g., ATI[N]) capturing amyloid, tau, synaptic loss, and inflammation better reflect disease complexity. Multi-omics integration—including genomics, proteomics, and single-nucleus RNA sequencing (snRNA-seq)—reveals glial subpopulations linked to inflammatory profiles (Sadick et al., 2022) and connects CSF proteomes to GWAS-identified risk variants (Kaiser et al., 2023; Tao et al., 2024). Artificial intelligence (AI) and machine learning (ML) handle high-dimensional datasets effectively. Deep learning (DL) models (e.g., InceptionV3 on ^18^F-FDG PET) achieve 0.98 AUC for early AD diagnosis (Jimenez-Mesa et al., 2023). ML identifies PD progression subtypes with 0.87–0.95 AUCs using serum NfL as a rapid progression marker (Dadu et al., 2022). Predictive models combining polygenic risk scores, plasma p-tau, and GFAP may stratify patients for targeted therapies. Liquid biopsies (e.g., plasma neuron-derived exosomes) and single-cell spatial transcriptomics promise novel, minimally invasive biomarkers.

### 4.5 Limitations

While this bibliometric analysis provides valuable insights into the trends in this field, several limitations should be acknowledged. As a bibliometric analysis, our findings are preliminary and hypothesis-generating rather than confirmatory. Further validation through clinical and experimental studies is warranted. First, our study relied exclusively on data from the WOS Core Collection, which, although comprehensive, may not capture all relevant research, particularly studies published in non-English languages or indexed in other databases like Scopus or PubMed. Second, our analysis focused solely on “Articles” and “Reviews,” potentially overlooking other important types of publications, such as conference papers or book chapters. Moreover, bibliometric results do not account for study quality, and citation metrics may be biased by factors such as journal visibility or open access status. Lastly, bibliometric analysis does not directly assess the quality of individual studies, and high citation counts may not necessarily reflect the scientific rigor or validity of the findings.

## 5 Conclusion

This bibliometric analysis has elucidated the research trends, hotspots, and future directions in the application of neural injury biomarkers for NDs. The findings highlight the increasing role of OS, NFTs, and neuroimaging technologies, particularly PET, in understanding disease mechanisms and advancing early diagnosis. Moving forward, researchers should prioritize multi-omics approaches and the validation of biomarkers to enhance clinical applicability and enable personalized treatment strategies in NDs.

## Data Availability

The original contributions presented in the study are included in the article/supplementary material, further inquiries can be directed to the corresponding author.
